# Epidemiology of Burn Injuries at a Newly Established Burn Care Center in Rasht

**DOI:** 10.5812/traumamon.6991

**Published:** 2012-10-10

**Authors:** Cyrus Emir Alavi, Seyed Hamid Salehi, Mohammad Tolouei, Koosha Paydary, Pirouz Samidoust, Mohammadreza Mobayen

**Affiliations:** 1 Department of Anesthesiology, Faculty of Medicine, Gilan University of Medical Sciences, Rasht, IR Iran; 2 Burn Research Center, Tehran University of Medical Sciences, Tehran, IR Iran; 3 Department of General Surgery, Faculty of Medicine, Gilan University of Medical Sciences, Rasht, IR Iran; 4 Students' Scientific Research Center (SSRC), Tehran University of Medical Science, Tehran, IR Iran

**Keywords:** Epidemiology, Burns, Iran

## Abstract

**Background:**

Advances in the care of burn injuries have resulted from the efforts of regional patient-based specialist teams at burn care centers.

**Objectives:**

We conducted this study to assess the four-year epidemiology of burn injuries in Rasht, Iran.

**Materials and Methods:**

In this cross-sectional study, medical records of 2274 burn patients, treated at Velayat hospital from January 2007 to December 2010 in Rasht, Iran, were assessed. Age, sex, level of education, occupation, severity and degree of burn, burn surface area, burn cause and outcome of patients were evaluated.

**Results:**

In our study the overall mortality rate was 8.7%; 65.7% of patients were men and 34.3% were women. Mean age of patients was 31.47 ± 22.67 years. Mean Total Burn Surface Area (TBSA) was 15.24 ± 18.4. Lowest TBSA was 0.5% and highest TBSA was 100%. Significant associations were observed between age (P = 0.0001), place of residence (P = 0.004), level of education (P = 0.0001), unemployment (P = 0.0001), marital status (P = 0.021), causes of burn (P = 0.0001), TBSA (P = 0.0001) and mortality rate. In our study, no significant difference was observed between age and sex (P = 0.071).

**Conclusions:**

Due to high prevalence of burn injuries in Iran, increasing the level of awareness of the society as well as adhering to safety procedures both at home and workplace is recommended via implementing effective national safety policies.

## 1. Background

Burn injury is one of the most important causes of morbidity, disability and death in the developing world that can affect psychosocial and functional aspects of patients. Nowadays, great advances have been made in the treatment and care of burn injuries globally and have resulted in increases in survival and quality of life (QOL) of burn patients (1). Such advances have primarily resulted from the efforts of regional patient-based specialist teams at burn care centers, highlighting the pivotal role of burn hospitals in providing appropriate care for patients in addition to defining the regional epidemiology of burn injuries (2).

Recent reports indicate that annual mortality rate of burn patients in developed countries is near 2.1 per 100000 people. In addition, current evidence suggests that national mortality rate is associated with cultural and socio-economic status of patients worldwide. While in US with 314 million inhabitants and 50 states, 128 burn care centers exist, this number is less than 10 in Iran with a population of 75 million people (3). In developed countries, the evident rise of LA50 (a percentage of total body burn that one out of two patients will survive in that particular center) from 50% in 1970 to 75% in 1997 has drawn attention toward improvement of QOL of patients as well as reducing complications such as hypertrophic scar and achieving better cosmetic outcomes (4, 5). Nonetheless in Iran, as well as other developing countries, reducing mortality rate of burn patients and the subsequent rise of LA50 is yet to be achieved as a primary goal, highlighting the needs for establishing more burn care centers in each povince of the nation (6). Established in 2007, Velayat burn care hospital is one of the newly established burn care centers in Rasht, Iran which provides advanced burn care services for patients in this region of the country. Until now, no previous study has been conducted to identify the characteristics and complications of burn injuries in Rasht.

## 2. Objectives

We conducted this study to assess the four-year epidemiology of burn injuries in this region of the country.

## 3. Materials and Methods

In this cross-sectional study, medical records of 2274 burn patients treated at Velayat hospital from January 2007 to December 2010 in Rasht, Iran were collected. Age, sex, level of education, occupation, severity and degree of burn, burn surface area, burn cause and outcome of patients were determined. The Institutional Review Board (IRB) of Tehran University of Medical Sciences approved the study protocol and checklists were filled out anonymously. All analyses were carried out using SPSS (for windows, version 16). Results were reported as mean ± standard deviation (SD) when normally distributed, otherwise as median and range. For comparing continuous data, the t-test and chi square test were performed. P-values less than 0.05 were considered statistically significant.

## 4. Results

**Figure 1:**
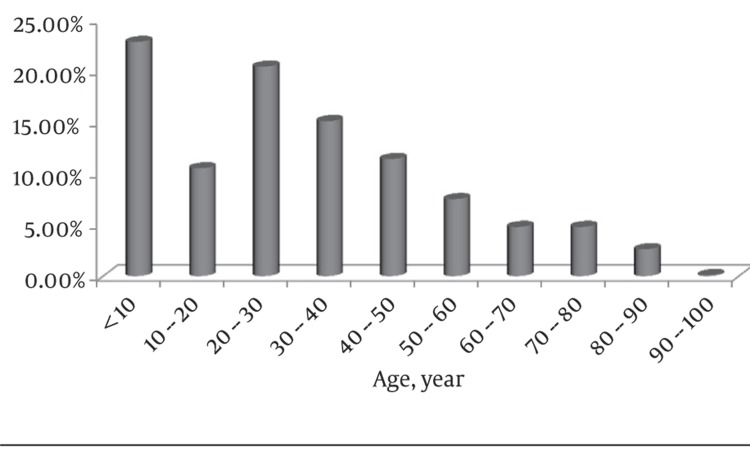
Age Distribution of Patients

**Figure 2:**
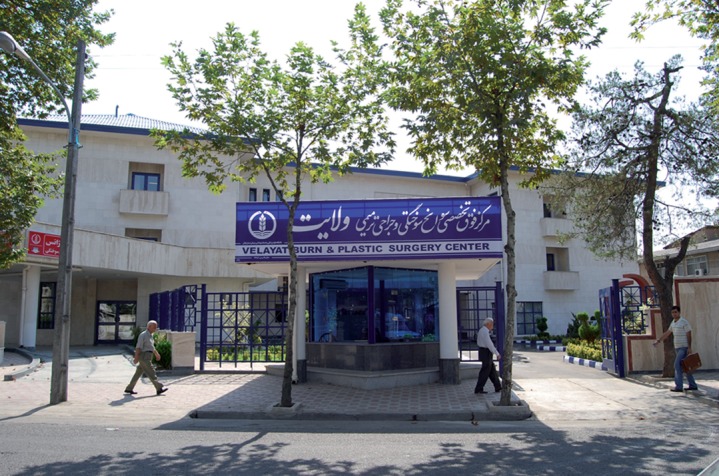
Velayat Burn Care Center, Rasht, Iran

**Figure 3:**
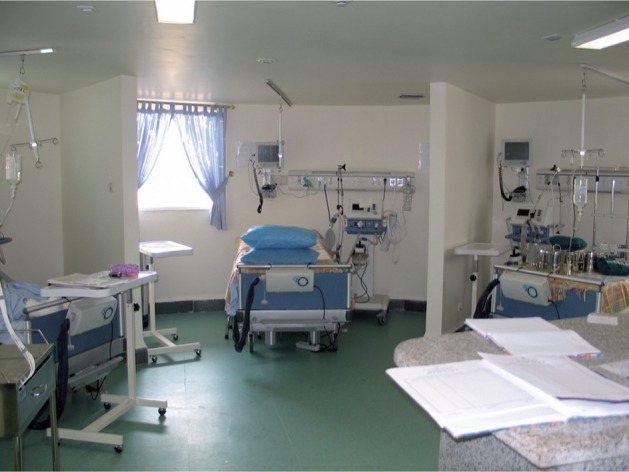
A Room With Several Beds, Velayat Burn Care Center, Rasht, Iran

In this study, Table 1 shows the year of admission, Total Burn Surface Area (TBSA) and length of hospitalization period of patients in addition to seasonal distribution and place of burn events were assessed. The overall mortality rate was 8.7% while65.7% of patients were men and 34.3% were women. Mean age of patients was 31.47 ± 22.67 years with a minimum and maximum age of one month and 92 years, respectively. Figure 1 shows the age distribution of patients. In the study population 64.1% were residents of urban areas and 35.9 % were residents of rural areas. Moreover, 18.7% of the hospitalized patients were illiterate, 16.1% had elementary education, 62.3% had diploma and only 2.9% of patients had academic education. Sixty seven percent of the patients were married and 33% were single. Most burns had occurred at home.(Figures 2 and 3)

**Table 1:** Year of Admission, Total Burn Surface Area (TBSA) and Length of Hospitalization Period of Patients in Addition to Seasonal Distribution and Place of Burn Events

	Distribution of patients according to the year of admission	
**Year**	N (%)	Mortality
**2007**	214 (9.4)	24 (11.2)
**2008**	497 (21.9)	64 (12.9)
**2009**	786 (34.6)	58 (7.4)
**2010**	777 (34.3)	51 (6.6)
**Total**	2274 (100)	197 (8.7)
	**Seasonal distribution of burns**	
**Season**	N, %	
**Spring**	478, 21	
**Summer**	526, 23.1	
**Fall**	623, 27.4	
**Winter**	647, 28.5	
**Total**	2274, 100	
	**Places of burn events**	
**Place**	N, %	
**Home**	1849, 81.3	
**Workplace**	262, 11.5	
**Others**	163, 7.2	
**Total**	2274, 100	
	**Distribution of TBSA in the study population**	
**TBSA**	N, %	
**< 25%**	1939, 85.3	
**25-50%**	207, 9.1	
**50-75%**	57, 2.5	
**75-100%**	71, 3.1	
**Total**	2274, 100	
	**Length of hospitalization**	
**Hospitalization (days)**	N, %	
**1-3**	514, 22.6	
**4-7**	690, 30.3	
**8 ≤**	1070, 47.1	
**Total**	2274, 100	

**Figure 4:**
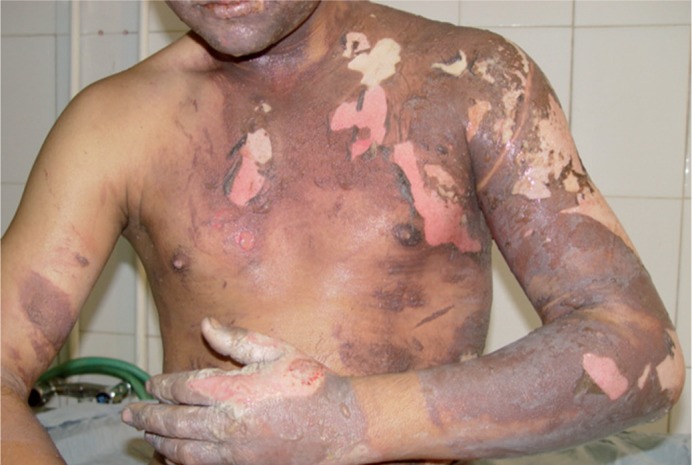
Burn Injury in an Adult Patient

**Figure 5:**
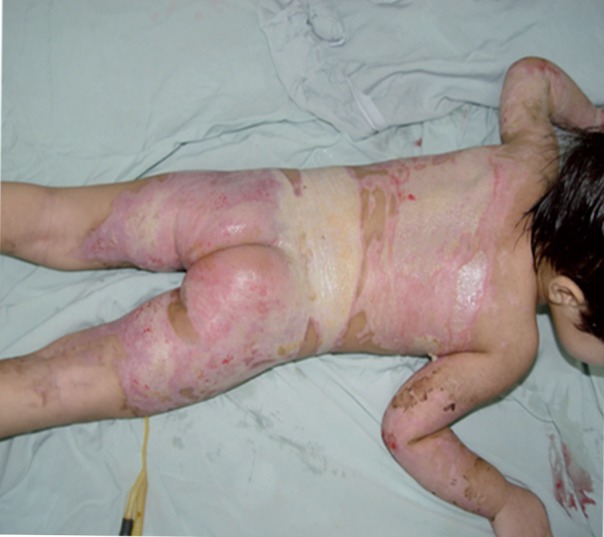
Burn Injury in a Child

Different causes of burn and mortality rate are outlined in Table 2. Almost all burns from various causes resulted in TBSA less than 25%, with the exception of self-inflicted burns that caused burns with TBSA of 75-100%. Mean TBSA was 15.24 ± 18.4. Lowest TBSA was 0.5% and highest TBSA was 100% (Table 1). Mean length of hospitalization was 9.21 ± 7.9 days, minimum length of stay was one day and maximum length of stay was 60 days (Table 1). According to the fact that our center was newly established, some patients with severe burn were referred to more equipped centers in Tehran; therefore, the average TBSA relatively decreased in our center.(Figures 4 and 5).

In the current study, we also meant to assess the possible associations between demographic/clinical features and mortality. A dramatic difference was observed in the mortality rate during different years of admission (P = 0.0001). Highest and lowest mortality rates were 12.9% in 2008 and 6.6% in 2010 respectively.

**Table 2:** Causes and Mortality Rate of Burn Injuries

	Female, N (%)	Male, N (%)	Mortality, N (%)	Total
**Flames**	136 (34.1)	263 (65.9)	66 (16.5)	399
**Petroleum, gasoline, alcohol**	44 (18.4)	195 (81.6)	31 (13)	239
**Hot liquids including hot water, tea, milk, foods**	429 (46.3)	497 (53.7)	15 (1.6)	926
**Tar, molten substances**	3(5)	57 (95)	0 (0)	60
**Explosion of cylinder, heater, stove, fireplace**	90 (24.5)	277 (75.5)	53 (14.2)	367
**Chemical burns with acid-base**	17 (40.5)	25 (59.5)	0 (0)	42
**Self-inflicted burns**	19 (46.3)	22 (53.7)	12 (29.3)	41
**Electrical burns**	7 (7.8)	83 (92.2)	3 (3.3)	90
**Hot substances, hot charcoal, exhaust**	32 (31.1)	71 (89.9)	0(0)	103
**Inhalation burns**	2 (100)	0 (0)	1 (50)	2
**Fireworks**	0 (0)	5 (100)	0 (0)	5
**Total**	779 (34.3)	1495 (65.7)	197 (8.7)	2274

There was no significant difference in the mortality rate among males and females (P = 0.071). Moreover, significant association was observed between age groups and mortality rate (P = 0.0001). Mortality rate in burn patients aged < 10 was 2.1% (11 cases out of 517 patients), while mortality rate in patients older than 50 was 14.4% (67 deaths out of 464 patients). The highest mortality rate was observed in the age group older than 90 years with 40% mortality (two deaths out of five patients). No relation was observed between season of admission and mortality (P = 0.147). Place of residence and mortality were statistically significant (P = 0.004). Compared to 7.3% deaths of patients living in urban areas, 11% of patients who lived in rural areas died.

Mortality was also statistically significant with regard to the level of education (P = 0.0001). The mortality rate was 5.6% in patients with academic education; while this rate reached to 19.5% in the illiterate. Moreover, unemployed were more prone to burn injuries as 62.4% of patients were unemployed. The mortality rate was higher in the unemployed patients; 12.4% in unemployed compared to 6.9% in employed patients (P = 0.0001). In the current study, populations with lower socioeconomic status were at higher risk of burn injuries. In this regard, 88.7% of burn patients were from low-to-moderate income families. The mortality rate was 0% in high and 10% in lower socioeconomic classes. This observation showed a borderline association (P = 0.066). Mortality was also statistically significant with marital status (P = 0.021); it was 11.5% in married patients compared to 8% in single patients. Additionally, there was a significant relation between various causes of burns and rate of mortality; for instance boiling water burn compared to other burn causes (P = 0.0001).

Sex differences observed in causes of burn injury were statistically significant (P = 0.0001). All causes of burns, except from inhalation , were more frequent in men. There was also statistically significant relation between causes of burns and TBSA (P = 0.02). Additionally, causes of burns and age of patients was statistically significant (P = 0.0001). Hot liquids were the most common causes in ages< 10 (46.7% of burns in age < 10 were due to hot liquids), while flames was the cause of burns in only 8.3% of this age group. Flames were the most common cause of burns in ages 20-40 (40.1%) followed by hot fluids, which accounted for 19.1 % of burns in this age group. Another significant association was observed between TBSA and mortality (P = 0.0001). Mortality rate was 1.6% in TBSA < 25%, while it was 91.5% in TBSA > 75%. However, the length of hospitalization and mortality did not show any association (P = 0.495).

## 5. Discussion

In this study, we evaluated the four-year epidemiology as well as the clinical outcomes of burn patients admitted at Velayat Burncare Center. Based on our records, the overall mortality rate was 8.7% and it had a statistically significant relation with higher age, living in rural areas, lower level of education, unemployment, being married and higher TBSA.

In our study, more than half of patients were male, which is similar to the studies undertaken in Yazd (central part of Iran), Turkey, Kuwait, Romania, Ireland, Brazil and Egypt (7-10). However, burns were more frequent in females in Shiraz (southern part of Iran), Yasuj (central part of Iran) and Kurdistan (western part of Iran) (11, 12). A review on the epidemiology of burns in low to moderate income countries reported that the incidence of burns is higher among males from birth to four years of age. After this age, the incidence substantially increased among females to levels higher than males (13). In addition, we did not find any significant association between sex and mortality, which is in contrast to reports from Ardabil (northwest part of Iran), Shiraz and Tehran in which women had higher mortality rates than men (14-16). Similar to other studies, burns were more common among 20-40 age groups and in children (7, 12, 14, 15, 17-21). The higher mortality in elderly patients and lower mortality in children is also consistent with previous reports (14). Similar to our observation, hot fluids were the most common cause of burns among children in previous studies (7, 9, 12, 14, 22).

In our study, there was no significant seasonal variation of burns requiring hospitalization. In contrast to reports from Shiraz (14, 17), Rasht has a moderate climate. Hence there is less need for heating cold months, a somewhat equal incidence of burns might be observed in such regions throughout the year. However, in other areas of the world with considerable seasonal variations in temperature such as in China, Egypt, Hong Kong and India, a higher incidence of burn injuries were reported during the cold months of the year (13). It is noteworthy to mention that the higher incidence of burn injuries and mortality rate among illiterates and less-educated patients of our study population is consistent with previous studies (23). Moreover, both hospitalization and mortality rate were more common among the unemployed. Considering that vast majority of burns occur at home, it seems reasonable that unemployed people who expend more time at home are at higher risk of burn.

Consistent with our findings, hot fluids were the most common cause of burn in Yazd, Kohgiluye-Boyerahmad and Tehran (8, 11, 21); however, flame was the most common cause of burns in Kurdistan, Sub-Saharan Africa, Bangladesh and Turkey (4, 6, 16, 22). In our study, flames, hot liquids, explosions, chemical burns, electrical burns and self-inflicted burns were more common in men; which is contrary to the reports from Shiraz, in which injuries caused by flames were more frequent among females (14). Self-inflicted burns have previously been reported in Ghana, India, Iran, Malawi, Nigeria, South Africa and Sri Lanka (13). India has a particularly high rate of self-inflicted burns among young women, whereas this rate is higher in middle-aged men in Europe (10). In addition, the higher prevalence of self-inflicted burns among males in our study is in contrast with the reports from Kurdistan and Kohgilue-Boyerahmad (11, 12). Even though flames were more fatal than other causes of burns in Tehran and Hamadan, we did not find any statistically significant difference between causes of burn and mortality (7, 9, 22).

In our study, 91.5% of patients with TBSA > 75% died, however mortality rate was only 1.6% in patients with TBSA < 25%. In a study undertaken in Tehran, TBSA was found to be one of the most predictive factors of patients' survival (16). Our findings also suggest that intentional burns are significantly related to higher TBSA. Considering higher mortality in higher TBSA, this result is similar to United States' national cohort of intentional burns, which reported that patients with intentional burns have higher morbidity and mortality rate (24). In terms of place of burn in our survey, the place in which burn has occurred did not alter the outcome which is similar to previous reports (6, 8, 9, 14, 15). This can be explained by the fact that the vast majority of burn victims are women and children who spend most of the day at home near stoves and heaters where flame and hot fluid-related burns occur. Additionally, rural areas have a higher incidence of burns which could be related to lower socioeconomic status and lower level of education (22). It is noteworthy to state that prior to the foundation of Velayat Hospital, burn patients from the northern Iran had to refer to other general hospitals or burn centers of other provinces located more than 300 kilometers away from Rasht.

This study however, had several limitations: Considering the retrospective design of the study, we were unable to further survey the complications of burn injuries. Moreover, the QOL of patients and the extent of which burn injury altered their life activities remains unknown. Therefore, we were unable to estimate the effects of establishing a new burn care center in northern part of Iran on national LA50 measures either.

In conclusion, our survey provides essential information on the epidemiology of burn injuries in northern Iran for further studies. Considering that homes were the most common place of burn injuries, national preventive interventions should focus on increasing the level of awareness of people by means of increasing safety measures at homes. The higher prevalence of burns among the illiterate and unemployed also highlights the fact educating people and employment may decrease regrettable burn events.
